# QTMP, a Novel Thiourea Polymer, Causes DNA Damage to Exert Anticancer Activity and Overcome Multidrug Resistance in Colorectal Cancer Cells

**DOI:** 10.3389/fonc.2021.667689

**Published:** 2021-05-28

**Authors:** Zhaoshi Bai, Qing Zhou, Huayun Zhu, Xinyue Ye, Pingping Wu, Lingman Ma

**Affiliations:** ^1^ Jiangsu Cancer Hospital & Jiangsu Institute of Cancer Research & the Affiliated Cancer Hospital of Nanjing Medical University, Nanjing, China; ^2^ School of Life Science and Technology, China Pharmaceutical University, Nanjing, China

**Keywords:** thiourea polymers, nanoparticles, DNA damage, multidrug resistance, apoptosis

## Abstract

Colorectal cancer (CRC) is one of the most common malignancies, and multidrug resistance (MDR) severely restricts the effectiveness of various anticancer drugs. Therefore, the development of novel anticancer drugs for the treatment of CRC patients with MDR is necessary. Quaternized thiourea main-chain polymer (QTMP) is a self-assembled nanoparticle with good water solubility. Notably, QTMP is not a P-glycoprotein (P-gp) substrate, and it exhibits potent cytotoxic activity against CRC cells, including HCT116/DDP and P-gp-mediated multidrug-resistant Caco2 cells. QTMP also exhibits a strong anticancer activity against SW480 cells *in vivo*. Interestingly, reactive oxygen species (ROS) and reactive nitrogen species (RNS) production were increased in a concentration-dependent manner in QTMP-treated HCT116, SW480 and Caco2 cells. Importantly, QTMP causes DNA damage in these CRC cells *via* direct insertion into the DNA or regulation of ROS and/or RNS production. QTMP also induces caspase-dependent apoptosis *via* overproduction of ROS and RNS. Therefore, QTMP is a promising anticancer therapeutic agent for patients with CRC, including those cancer cells with P-gp-mediated MDR. The present study also indicates that the design and synthesis of anticancer drugs based on thiourea polymers is promising and valuable, thereby offering a new strategy to address MDR, and provides reference resources for further investigations of thiourea polymers.

## Introduction

Colorectal cancer (CRC) is the third leading cause of new cancer cases and the second most frequent cause of cancer-related death (1). Despite considerable research efforts aimed at developing novel therapeutic approaches against CRC, the frequent occurrence of multidrug resistance (MDR) severely restricts the effectiveness of various anticancer drugs (2, 3). Therefore, the development of novel promising and effective anticancer drugs for the treatment of CRC patients with MDR is necessary.

MDR is a well-defined phenomenon constituting combined resistance of cancer cells to several anticancer drugs following exposure to one drug (4, 5). MDR is often associated with the overexpression of P-glycoprotein (P-gp), which is a member of the ATP-binding cassette (ABC) family of efflux transporters, which escalate the efflux of anticancer drugs from cancer cells and lead to a decrease in intracellular drug concentrations (6, 7). Coadministration of a P-gp inhibitor and a primary chemotherapeutic drug is an effective approach to overcome P-gp-mediated MDR (5, 7, 8). Unfortunately, no P-gp inhibitors are approved for clinical use due to their poor potency, unsatisfactory toxicity, and low selectivity (9). Importantly, the development of a new anticancer drug that is a poor substrate of P-gp would also offer an effective strategy against P-gp-mediated MDR.

Thioureas are a class of sulfur-containing organic compounds and have the general formula (R_1_R_2_N)(R_3_R_4_N)C=S. These compounds act as pharmacophores in many biologically active molecules (10, 11). One of the most important applications of thioureas and their derivatives is their anticancer activity (12, 13). Polymers are increasingly used as nanocarriers to encapsulate or conjugate anticancer agents (14, 15). Notably, a few synthetic biopolymers have some intrinsic pharmaceutical activities (16, 17). Polymers are particularly advantageous as macromolecular drugs in nanoscale hydrodynamic sizes, which may allow long blood-circulation times, greater tumor accumulation and high selectivity and activity (18–20). Polymers are difficult substrates of P-gp because of their special structure and large molecular weight and remain effective against cancer cells with P-gp-mediated MDR (21–23). Therefore, it is promising and valuable to design and synthesize anticancer drugs based on thiourea polymers.

Our research group designed, synthesized and screened a series of novel thiourea polymers, and found that quaternized thiourea main-chain polymer (QTMP) exhibited strong cytotoxic activity against various CRC cells, including multidrug-resistant cells. However, the detailed mechanisms remain unclear. Therefore, further investigation of the anticancer mechanisms of QTMP against CRC cells is needed. It will provide a theoretical basis for the use of QTMP as a promising anticancer drug in the treatment of CRC, including for patients with MDR.

## Materials and Methods

### Chemical Compounds and Reagents

1,6-Hexanediamine (92%), N,N-bis(3-aminopropyl)methylamine (98%), chloroform (99%), sodium hydroxide (50 wt% aqueous solution) and benzyltriethylammonium chloride (TEBAC) were purchased from Wanqing Chemical Instrument Co., Ltd (Nanjing, P.R.C.). Paclitaxel, oxaliplatin, cisplatin (DDP), verapamil (VRP) and z-VAD-FMK were obtained from Selleck Chemicals (Houston, USA). Annexin V-FITC/PI double-staining kit was provided by KeyGen (Nanjing, P.R.C.). N-acetyl-L-cysteine (NAC), hemoglobin, Hoechst 33342, crystal violet, 4’,6-diamidino-2-phenylindole (DAPI), 3-amino-4-aminomethyl-2’,7’-difluorescein diacetate (DAF-FM DA) and 2’,7’-dichlorofluorescein (DCFH-DA) were provided by Beyotime Biotechnology (Nanjing, P.R.C.). Propidium iodide (PI), calf thymus DNA (ct-DNA), 3-(4,5-dimethylthiazol-2-yl)-2,5-diphenyl-tetrazolium bromide (MTT) and ethidium bromide (EB) were purchased from Sigma-Aldrich (St. Louis, USA). γ-H2AX (phospho S139) antibody was obtained from Abcam (Cambridge Science Park, Cambridge, UK). Primary antibodies against specific for cleaved caspase-3, cleaved caspase-9, Bax, poly (ADP-ribose) polymerase (PARP), β-actin and horseradish peroxidase (HRP)-conjugated secondary antibodies were purchased from Cell Signaling Technology (Boston, USA).

### Synthesis of Monomeric 1,6-Diisocyanohexane (DICH)

DICH was synthesized as described previously (24). 1,6-Hexanediamine (100 mmol) and TEBAC (2.2 mmol) were dissolved in chloroform (260 mmol), and sodium hydroxide (60 ml, 50 wt%) was slowly added dropwise into the vigorously stirred solution. The reaction was stirred overnight at 40°C. Subsequently, the solution was cooled to room temperature and extracted with dichloromethane (50 ml) three times. The organic layer was purified by passing through silica gel with an eluent of a petroleum ether/CH_2_Cl_2_ mixture (v/v, 1/1). The product was dried in a vacuum oven at 40°C for 8 h. ^1^H NMR (600 MHz, chloroform-D, **Figure S1**), δ (TMS, ppm): 3.32 (t, 4H), 1.72 (t, 4H), 1.48 (m, 4H).

### Synthesis of the Thiourea Main-Chain Polymer (TMP)

The procedure for TMP synthesis was reported previously (24). DICH (1 mmol), elemental sulfur (4 mmol), and N,N-bis(3-aminopropyl) methylamine (1 mmol) were added into a Schlenk flask under nitrogen. A mixture of degassed solvent (DMF:toluene = 1:2, 1 ml) was injected into the reaction vial. The flask was heated to 70°C for 15 h to complete the polymerization reaction. Subsequently, the reaction mixtures were precipitated in methanol. Solids were collected and dried to obtain the final polymers. The molecular weight was characterized by gel permeation chromatography (GPC).

### Quaternization of the TMP

The TMP was quaternized *via* the following procedure. Specifically, the main-chain polymer (1 g) was mixed with an excess of iodomethane (2 g) in DMF. The mixture was reacted at room temperature overnight and was then precipitated into dimethyl ether to obtain brown solids.

### Morphology and Size Distribution of QTMP

A diluted QTMP solution (10 mg/ml) was added dropwise onto a carbon-coated copper grid and negatively staining with 2% (w/v) phosphotungstic acid. The morphology of QTMP was examined by a JEOL 1230 transmission electron microscope (TEM).

The size, polydispersity index (PDI) and zeta potential of QTMP were measured using a Zetasizer (Nano-ZS90, Malvern Co., U.K.).

### Cell Culture

The human cancer cell lines SW480, HCT116 and CaCO_2_ were purchased from the Shanghai Institute of Cell Resource Center Life Science (Shanghai, P.R.C.). HCT116/DDP cells were purchased from Hunan Fenghui Biotechnology Co., Ltd (Changsha, P.R.C.). The cells were subjected to short tandem repeat analysis and validated as free from mycoplasma contamination. Cells were used within 6 months. All cells were cultured in Dulbecco’s Modified Eagle’s Medium (DMEM) supplemented with 10% fetal bovine serum (FBS, CellMax), penicillin and streptomycin at 37°C in a humidified atmosphere with 5% CO_2_ and were maintained in logarithmic growth phase for all experiments. HCT116/DDP cells were periodically cultured in 5 μg/ml DDP to maintain the DDP resistant phenotype and shifted into DDP-free medium for 1 week before use.

### MTT Assay

Cell viability was determined by a routine MTT assay (25). In brief, cells were seeded in 96-well plates at 3500 cells/well and treated with medium or different concentrations of QTMP (0~100 μg/ml) for 72 h. The MTT solution (5 mg/ml) was added to each well and incubated for 4 h at 37°C. The MTT was removed, and DMSO was added to each well. The absorbance was measured at 492 nm using a microplate reader (MK3, Thermo, Germany). The IC_50_ values were calculated using Dose-Effect Analysis with Microcomputers software.

### Anticancer Effects *In Vivo*

Manipulations of animals were performed in accordance with the Guideline for the Care and Use of Laboratory Animals published by the US National Institutes of Health (NIH Publication No. 85-23, revised 1996), and was approved by the Experimental Animal Ethic Committee of China Pharmaceutical University and the Science and Technology Department of Jiangsu Province (2021-03-001). Xenograft tumors were generated in 5-week-old male BALB/c nude mice (Laboratory Animal Center of Yangzhou University, Yangzhou, P.R.C.) using SW480 cells (5 × 10^6^ cells/mice). The tumor volumes were calculated from caliper measurements using the following formula: (length × width^2^)/2. When tumor volumes reached about 100 mm^3^, mice were randomized into 3 groups: model (saline); oxaliplatin (10 mg/kg, oxaliplatin injection diluted in saline); and QTMP (20 mg/kg, dissolved in saline). Mice were intraperitoneally administered every 2 days for 12 days. All tumors and organs were collected and measured at the end of the experiment.

### Crystal Violet Staining

Cells were seeded into six-well plates at a density of 5000 cells/well and treated with different concentrations of QTMP (5~20 μg/ml) for 48 h. Cells were shifted into drug-free fresh medium for two weeks and were then fixed with 4% paraformaldehyde, stained with crystal violet for 40 min and photographed with a digital camera (26, 27).

### Cell Cycle Analysis

Cells were seeded into six-well plates at a density of 2×10^5^ cells/well. Cells were incubated with different concentrations of QTMP (final concentrations ranging from 5 to 20 μg/ml) for 48 h, collected by centrifugation and fixed in 70% (v/v) ice-cold ethanol. After washing with ice-cold PBS for twice, cells were stained with PI (50 μg/ml) containing RNase A at 4°C for 30 min. The samples were detected by fluorescence-activated cell sorting (FACS) (26).

### Changes in Nuclear Morphology

Cells (1×10^4^ cells/well) were seeded in 24-well plates, grown overnight, and then treated with QTMP (10 μg/ml) for 48 h. Cells were fixed in 4% paraformaldehyde for 15 min and incubated with Hoechst 33342 for 30 min. After washing off the unbound dye, the stained cells were imaged under a fluorescence microscope (Olympus, Japan).

### Apoptosis Assay

Annexin V-FITC/PI double-staining was used to detect cell apoptosis (23). Cells were seeded into six-well plates at a density of 2×10^5^ cells/well and treated with different concentrations of QTMP (5~20 μg/ml) for 48 h. The collected cells were stained using Annexin V-FITC and PI double-staining kits. Samples were analyzed using FACS.

### Measurement of ROS and RNS

DCFH-DA and DAF-FM DA were used to detect the accumulation of intercellular ROS and RNS, respectively (28, 29). Specifically, cells were treated with QTMP (5~20 μg/ml) for 48 h and were then incubated with DCFH-DA or DAF-FM DA for 40 min at 37°C in the dark. Cells were then washed with ice-cold PBS, and the fluorescence intensity was analyzed by FACS.

### DNA Binding Experiments

Competitive binding assays were performed using fluorescence spectroscopy to investigate the binding between QTMP and DNA (25). In brief, a mixture of ct-DNA (100 μg/ml) and EB (5 μM) was incubated in the dark at 37°C for 10 min and different concentrations of QTMP (0~25 μg/ml) were added. The fluorescence emission spectra in the range of 500~600 nm at an excitation wavelength of 525 nm were recorded.

The ct-DNA samples were mixed with isovolumetric QTMP (0~25 μg/ml) and subjected to a 15-min incubation at room temperature. The DNA shift was detected by 1% (w/v) agarose gel electrophoresis under UV illumination in a gel imaging system (Bio-Rad).

### Western Blot Analysis

Western blot analysis was performed according to previous procedures (25, 26, 28). Protein samples were prepared in ice-cold RIPA buffer supplemented with protease and phosphatase inhibitors. Protein concentrations were measured using a BCA protein assay (30). Cell lysate proteins were separated on SDS-polyacrylamide gels and transferred to PVDF membranes. Membranes were blocked with 5% BSA or nonfat milk and were then sequentially probed with primary antibodies overnight at 4°C and incubated with HRP-conjugated secondary antibodies for 2 h at room temperature. Signals were visualized by enhanced chemiluminescence, and densitometric analysis was performed using ImageJ2 software.

### Statistical Analysis

Data are presented as the means ± SDs, and all experiments were performed in triplicate. Comparisons between groups were performed with one-way ANOVA followed by Tukey’s *post hoc* test or unpaired Student’s *t*-test using SPSS 22.0 software. A *p*-value < 0.05 was considered to indicate a significant difference.

## Results

### Synthesis of QTMP

**Figure 1:**
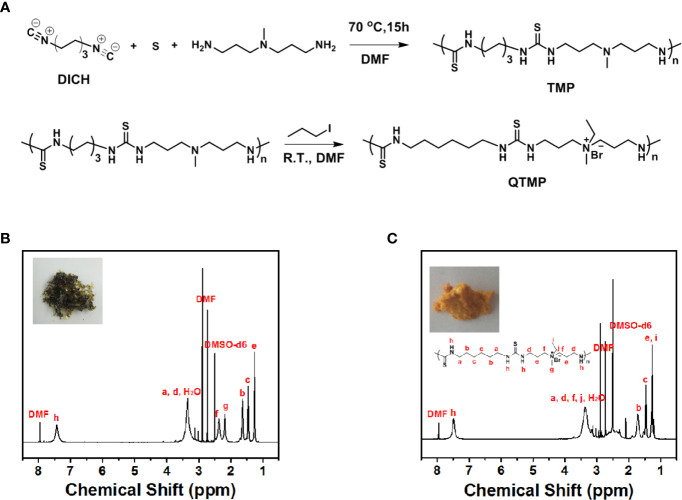
Synthesis of QTMP. **(A)** Synthesis scheme for polymer TMP and QTMP. **(B)** ^1^H NMR spectrum for polymer TMP. The inset shows an image of the synthesized polymer. **(C)** ^1^H NMR spectrum for the polymer QTMP. The inset shows an image of the synthesized polymer.

The TMP was conveniently prepared *via* three-component polymerization (**Figure 1A**). The ^1^H NMR and GPC spectra indicated successful polymer synthesis [^1^H NMR (600 MHz, DMSO-d_6_), δ (TMS, ppm): 7.5 (s, 2H), 3.5 (m, 4H), 2.4 (m, 4H), 2.2 (m, 3H), 1.7 (m, 4H), 1.7 (m, 4H), and 1.2 (e, 4H), **Figure 1B**]. The molecular weight of TMP was approximately 6 kDa (**Figure S2**). The molecular weight polydispersity detected by GPC was approximately 4.2, which was much higher than that of polymers generated by standard controlled polymerization, indicating the rational step-growth mechanism of the polymerization (**Figure S2**).

To improve the solubility and affinity for biomembranes, quaternization was used to obtain the quaternary ammonium groups on the polymer main chain, as shown in **Figure 1A** (below panel). TMP (*M*w > 2000 Da) was quaternized with iodomethane to obtain a positive charge at the amine position [^1^H NMR (600 MHz, DMSO-d_6_, **Figure 1C**), δ (TMS, ppm): 7.5 (s, 2H), 3.5 (m, 6H), 2.4 (m, 4H), 2.2 (m, 3H), 1.7 (m, 4H), 1.7 (m, 4H), and 1.2 (e, 7H)]. Notably, new peaks for methylene and methyl groups at 3.5 and 1.5 ppm were observed, indicating successful polymerization. This process resulted in a high water solubility of QTMP, which was suitable for biomedical use.

### Characterization of QTMP

**Figure 2:**
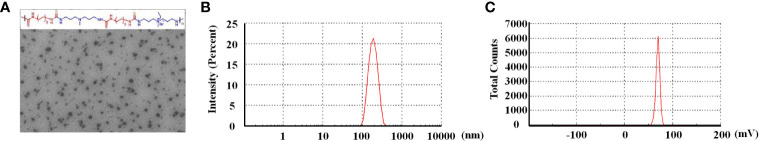
Characterization of QTMP. **(A)** Representative transmission electron micrograph of QTMP (scale bar = 200 nm). **(B)** Particle size distribution in PBS, as detected by DLS. **(C)** Zeta potential of QTMP.

A representative TEM (**Figure 2A**) showed that QTMP was nearly spherical with a smooth shape devoid of agglomeration. The size distribution of QTMP with a homogeneous particle diameter, was approximately 162.7 ± 14.6 nm (**Figure 2B**), and its PDI, as detected by DLS, was 0.26 ± 0.04 in PBS (**Table 1**). The zeta potential of QTMP was measured in water as 23.96 ± 5.20 mV (**Figure 2C**). These data indicate that QTMP is a cationic polymer with a uniform distribution in solution.

**Table 1:** Characterization of QTMP (mean ± SD, n = 3).

Solvent	Particle size (nm)	Polydispersity index	Zeta potential (mV)
Water	164.5 ± 12.4	0.39 ± 0.02	23.96 ± 5.20
PBS	162.7 ± 14.6	0.26 ± 0.04	Not applicable
DMEM+10%FBS	171.4 ± 17.8	0.42 ± 0.07	Not applicable

### QTMP Exhibits Potent Cytotoxic Activity Against Sensitive and Drug-Resistant CRC Cells *In Vitro* and *In Vivo*

**Figure 3:**
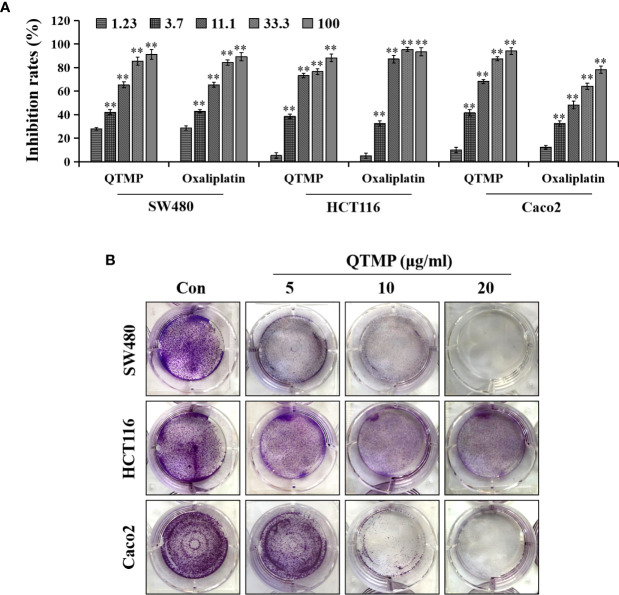
QTMP suppresses CRC cell proliferation. **(A)** The proliferation inhibitory effects of QTMP (0~100 μg/ml) on SW480, HCT116 and Caco2 cells were detected by an MTT assay. **(B)** The growth inhibitory effects of QTMP (5, 10, 20 μg/ml) on these CRC cells were measured by crystal violet staining. **p < 0.01 *vs* control.

First, we evaluated the cytotoxic activity of QTMP against CRC cells *in vitro*. MTT assays showed that QTMP inhibited the proliferation of SW480, HCT116 and Caco2 cells in a concentration-dependent manner (**Figure 3A**). Notably, SW480, HCT116 and Caco2 cells are commonly used cell culture models for the study of CRC *in vitro* (3, 25). Caco2 cells express high levels of P-gp protein, and these cells are naturally multidrug-resistant (25, 31, 32). The IC_50_ values of QTMP in SW480, HCT116 and Caco2 cells were 4.73 ± 1.12, 5.66 ± 1.32 and 6.14 ± 1.45 μg/ml, respectively, indicating only slight differences in the IC_50_ values of QTMP in these CRC cell lines (**Table 2**). Interestingly, QTMP also exhibited a strong anticancer activity against HCT116/DDP cells (DDP-resistant HCT116 cells), with an IC_50_ value of 5.34 ± 1.23 μg/ml (**Figure S3**). Crystal violet staining showed that the QTMP-treated CRC cells lost the capacity to proliferate in a concentration-dependent manner (**Figure 3B**).

**Table 2:** IC_50_ of QTMP against various human cancer cell lines (mean ± SD, n = 3).

Cell line	IC_50_ (μg/ml)		
	Paclitaxel	Oxaliplatin	QTMP
SW480	45.21 ± 4.21	4.67 ± 1.37	4.73 ± 1.12
HCT116	35.58 ± 5.41	7.78 ± 1.48	5.66 ± 1.32
Caco2	1800.45 ± 34.24	13.81 ± 2.68	6.14 ± 1.45

SW480 cell-bearing mice treated with QTMP or oxaliplatin showed an attenuated tumor growth compared to mice in the model group (**Figure S4A**). The overall size and weight of tumors in the QTMP- and oxaliplatin- treated groups were significantly lower than those of model group (**Figures S4B, C**). In addition, we did not observe significant toxicity of QTMP reflected by the loss of body weight (**Figure S4D**). Viscera index analyses (i.e., the ratio of the visceral weight to body weight [mg/g]) revealed no differences in the hearts, livers, lungs or kidneys between the QTMP and model groups, suggesting that this compound has low toxicity (**Figure S4E**).

Taken together, these data suggest that QTMP treatment exhibits strong anticancer activity against both sensitive and drug-resistant CRC cells *in vitro* and *in vivo*.

### QTMP Induces Apoptosis in CRC Cells

**Figure 4:**
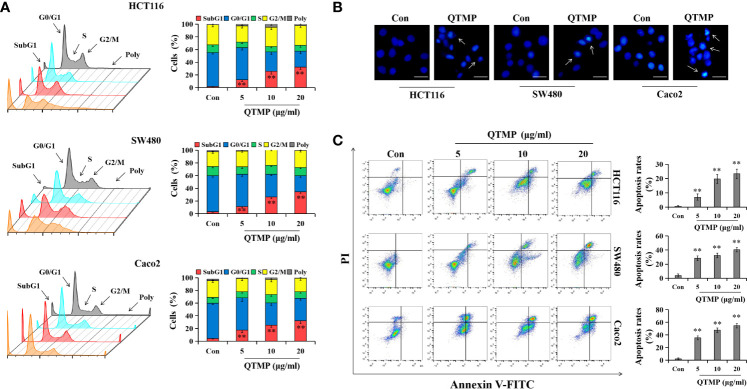
QTMP induces apoptosis in CRC cells. **(A)** FACS analysis for cell cycle distribution after treatment with QTMP (5, 10, 20 μg/ml) for 48 h in SW480, HCT116 and Caco2 cells. The original pictures (left); quantification of cell percentages in specific cell cycle phases (right). **(B)** Nuclear morphological changes in CRC cells exposed to QTMP (10 μg/ml) for 48 h were visualized by Hoechst 33342 staining. White arrows: apoptotic cells. (scale bar = 100 μm). **(C)** CRC cells treated with QTMP (5, 10, 20 μg/ml) for 48 h were stained with Annexin V-FITC/PI double-staining kits to measure apoptotic cells; quantification (right). **p < 0.01 *vs* control.

Most anticancer drugs cause cell cycle arrest and trigger apoptosis to achieve their effects (33, 34). The results of the cell cycle assay (**Figures 4A** and **S5A**) demonstrated that QTMP did not cause cell cycle arrest in the CRC cell lines. However, notable apoptosis was observed in the CRC cells after incubation with QTMP, and an apoptotic peak (the proportion of sub-G1 cells) appeared in the DNA histogram of the FACS data. The apoptosis rate of cells increased in a concentration-dependent manner (**Figures 4A** and **S5A**).

Subsequently, Hoechst 33342 staining revealed morphological changes that were characterized by condensed chromatin and fragmented nuclei in QTMP-treated CRC cells, indicating the appearance of apoptosis (**Figure 4B**). Notably, Annexin V-FITC/PI double staining also showed that QTMP induced apoptosis in SW480, HCT116, HCT116/DDP and Caco2 cells (Annexin-V-positive) in a concentration-dependent manner (**Figures 4C** and **S5B**). These data indicate that QTMP exerts its strong anticancer effects by inducing apoptosis in these CRC cells.

### QTMP Causes Caspase-Dependent Apoptosis in CRC Cells

**Figure 5:**
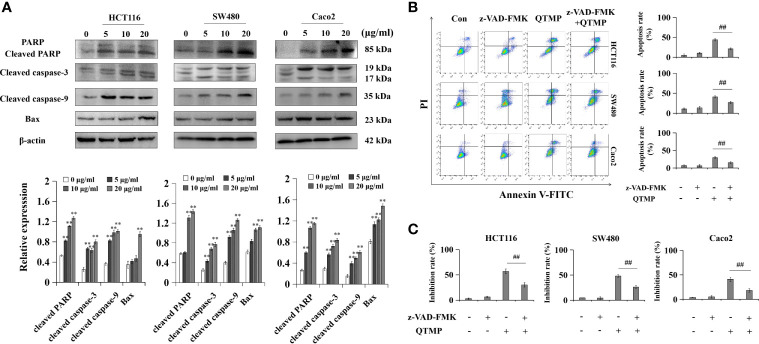
QTMP causes caspase-dependent apoptosis in CRC cells. **(A)** The expression levels of apoptosis-related proteins in SW480, HCT116 and Caco2 cells treated with QTMP (5, 10, 20 μg/ml) for 48 h; quantification (lower panel). **(B, C)** Annexin V-FITC/PI double-staining or MTT assay for CRC cells pretreated with z-VAD-FMK (50 mM) or vehicle for 3 h and continually incubated with QTMP (10 μg/ml) for another 48 h; quantification (right). **p < 0.01 *vs* control; ^##^ *p* < 0.01 *vs* QTMP alone.

To further clarify the underlying anticancer mechanisms of QTMP, the expression of apoptosis-related proteins was examined by western blot analysis using lysates of these CRC cells. As shown in **Figure 5A**, QTMP significantly upregulated the expression of Bax, cleaved PARP, cleaved caspase-3 and cleaved caspase-9 in a concentration-dependent manner in SW480, HCT116 and Caco2 cells. Caspases are a family of cysteine proteases that are widely known as the principal mediators of the apoptotic cell death response (35). To further clarify whether caspases were involved in QTMP-induced apoptosis, the proportion of apoptotic cells in the presence or absence of the pan-caspase inhibitor z-VAD-FMK was measured. As shown in **Figure 5B**, z-VAD-FMK significantly downregulated QTMP-induced apoptosis in these CRC cells. The MTT results also indicated that z-VAD-FMK significantly decreased QTMP-induced inhibition of cell proliferation in SW480, HCT116 and Caco2 cells (**Figure 5C**). These data suggest that QTMP treatment-induced apoptosis is caspase-dependent in these CRC cells.

### QTMP-Induced Apoptosis Depends on ROS and RNS in CRC Cells

**Figure 6:**
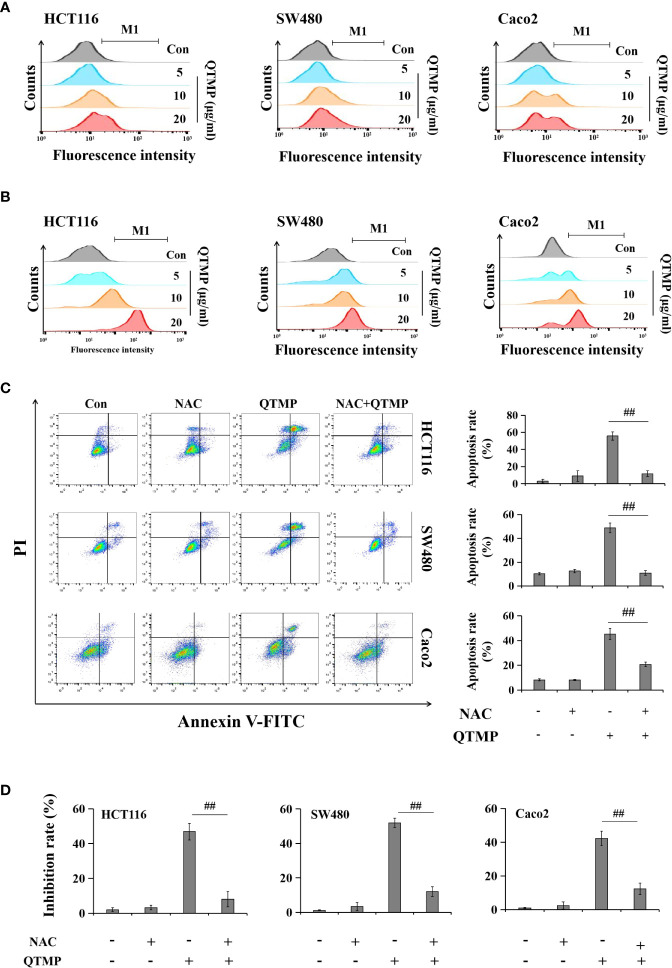
QTMP-induced apoptosis is dependent on ROS and RNS in CRC cells. **(A, B)** DCFH-DA and DAF-FM DA staining for ROS and RNS detection, respectively, in CRC cells treated with QTMP (5, 10, 20 μg/ml). **(C)** Annexin V-FITC/PI double-staining for cells pretreated with NAC (5 mM) or vehicle for 1 h and continually incubated with QTMP (10 μg/ml) for another 48 h. **(D)** The inhibitory rates of cell proliferation were measured by an MTT assay. SW480, HCT116 and Caco2 cells pretreated with NAC (5 mM) or vehicle for 1 h were continually incubated with QTMP (10 μg/ml) for another 48 h. **p < 0.01 *vs* control; ^##^ *p* < 0.01 *vs* QTMP alone.

The overaccumulation of ROS and RNS creates oxidative and nitrosative stress and plays important roles in regulating cell survival and death (36, 37). As shown in **Figures 6A** and **S6**, QTMP induced ROS generation in a concentration-dependent manner, as indicated by a rightward shift in fluorescence in the FACS plots in SW480, HCT116 and Caco2 cells. Similarly, RNS production was increased in a concentration-dependent manner in QTMP-treated CRC cells (**Figures 6B** and **S7**).

To further investigate the functional involvement of ROS in QTMP-induced apoptosis, the ROS scavenger NAC was used. As expected, NAC significantly reduced QTMP-induced apoptosis in SW480, HCT116 and Caco2 cells (**Figure 6C**). Pretreatment with NAC also attenuated the inhibitory effects of QTMP on the proliferation of CRC cells (**Figure 6D**). Hemoglobin (a NO scavenger) also markedly suppressed the apoptosis and proliferation inhibition induced by QTMP in these CRC cells (**Figures S8** and **S9**). These results indicate that QTMP-induced apoptosis depends on ROS and RNS production in these CRC cells.

### QTMP Directly and Indirectly Induces DNA Damage in These CRC Cells

**Figure 7:**
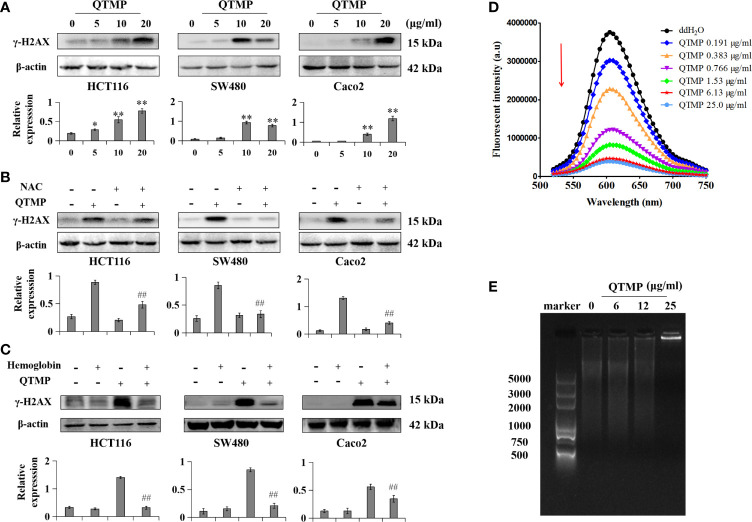
QTMP-induced DNA damage in CRC cells. **(A)** QTMP (5, 10, 20 μg/ml) upregulation of γ-H2AX (Ser139) protein expression was analyzed by western blotting; quantification (lower panel). **(B, C)** Western blot assay for the expression of γ-H2AX (Ser139) in cells pretreated with NAC (5 mM) or hemoglobin (20 μM) for 1 h and continually incubated with QTMP (10 μg/ml) for another 48 h; quantification (lower panel). **p < 0.01 *vs* control; ^##^ *p* < 0.01 *vs* QTMP alone. **(D)** The competitive binding of QTMP and EB to ct-DNA was determined by a fluorescence spectrophotometer (λex = 525 nm, λem = 600 nm). **(E)** Gel retardation assay for DNA binding detection.

The accumulation of intracellular ROS and RNS may cause DNA damage, which ultimately activates apoptosis (36, 37). QTMP induced DNA damage in CRC cells by significantly increasing the expression of γ-H2AX (Ser139) (**Figure 7A**). Interestingly, NAC and hemoglobin decreased the QTMP-induced upregulation of γ-H2AX expression (**Figures 7B, C**).

Importantly, QTMP is positively charged, and DNA is negatively charged (38). The fluorescence emission spectra of the ct-DNA-EB complex were weakened regularly at 600 nm with increasing concentrations of QTMP, but the shape of the fluorescence peak did not change (**Figure 7D**). In addition, the agarose gel electrophoresis results showed that ct-DNA-QTMP complex samples were increasingly retained at the starting position with increasing QTMP concentrations (**Figure 7E**). Collectively, these data suggest that QTMP causes DNA damage indirectly by increasing ROS and RNS production and directly by binding to DNA.

### QTMP Is Not a Substrate for Efflux Transporters

**Figure 8:**
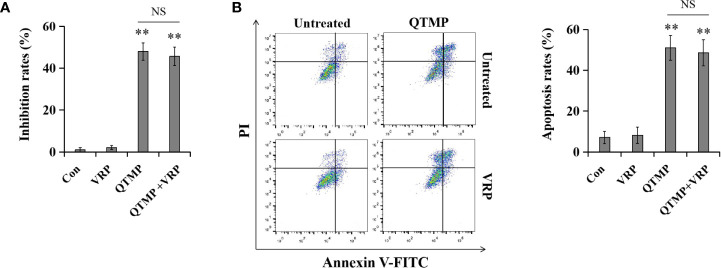
QTMP is not a substrate for efflux transporters. Cells were treated with QTMP (10 μg/ml) alone or in combination with VRP (10 mM) for 72 or 48 h prior to the MTT assay **(A)** or Annexin V-FITC/PI double-staining **(B)**.

MDR of Caco2 cells is due to the high expression of P-gp. To determine whether QTMP is a substrate of P-gp, the P-gp inhibitor VRP was used to inhibit the function of P-gp in Caco2 cells. As shown in **Figure S10A**, the MTT assay showed that VRP significantly strengthened the growth inhibitory effect of paclitaxel, a typical substrate of P-gp. Notably, VRP did not affect the inhibitory effect of QTMP in Caco2 cells (**Figure 8A**). Moreover, VRP dramatically increased the paclitaxel-induced apoptosis rate but not the QTMP-induced apoptosis rate in Caco2 cells (**Figures 8B** and **S10B**). Collectively, these findings suggest that QTMP is not a P-gp substrate.

## Discussion

Colorectal cancer remains a global health problem and one of the leading causes of cancer-related deaths (1, 2). MDR develops in most patients with colorectal cancer, which limits the therapeutic efficacies of many conventional anticancer drugs. Nanoparticles improve the targeting of cancer therapy *via* the inherent passive targeting phenomena and the adoption of active targeting strategies (39, 40). Nanocarriers also encapsulate the drug to protect it against degradation, which enhances its bioavailability for therapeutic application (41). So, nanomaterials with intrinsic anticancer activity will be more useful for the therapy of cancers, including MDR colorectal cancer.

In this study, a thiourea polymer (i.e., QTMP) was designed and synthesized by our group. Hereinto, QTMP self-assembled into nearly spherical nanoparticles with a smooth shape that was devoid of agglomeration. Further, QTMP exhibited very similar average diameters in different solvents, including water, PBS and DMEM/10% FBS. Importantly, QTMP not only had good water solubility but also had significant anticancer activity against various CRC cells, including multidrug-resistant cells. Notably, carbon-sulfur bond (259 kJ/mol) is less stable than common covalent bonds, such as carbon-carbon (347 kJ/mol) and carbon-oxygen single bond (358 kJ/mol) (42, 43); however, the bonding energy for C-S single bond is comparable with many classical covalent bonds including C-Br (276 kJ/mol), Cl-Cl (239 kJ/mol) and N-O (201 kJ/mol) (44), indicating that there has a relatively stable covalent bond between C and S and the C-S bond-breaking is not that easy. Considering the biological use of the thioether polymer (C-S), QTMP may be stable under mild conditions (body temperature without highly reactive chemicals, such as high temperature or strong oxidant). Thus, in this design, the high efficacy of QTMP against CRC cells may be partially attributed to its targeting and biological activities as a nanoparticle. However, a detailed and clear explanation of the mechanism is lacking.

Subsequently, a detailed and clear explanation of the mechanism underlying QTMP against CRC cells, including drug-resistant CRC cells, was studied. MDR is considered a multifactorial response that is determined by various interdependent and independent biomechanistic pathways and can be mediated *via* various mechanisms (45, 46). Induction and activation of ABC transporters (including ABCB1, ABCG2, ABCC1, ABCC10 and others) is the most common contributor to MDR (47, 48). Notably, regarding these efflux transporters, the most extensively studied and clinically prevalent problem is the overexpression of ABCB1, which is also called P-gp (49). In addition, P-gp overexpression caused by the unique physiological structure of the colorectum has been identified as the major and common factor of MDR in CRC cells (47, 50). Therefore, P-gp function inhibition or P-gp avoidance are the main strategies to solve MDR resulted from P-gp overexpression (47, 51). Unfortunately, there are no clinically available P-gp inhibitors to reverse MDR in cancer patient (7, 52). VRP is a calcium channel blocker that was intended for use in the treatment of hypertension and angina for decades, and it is a P-gp inhibitor that reverses MDR caused by ABCB1 *in vitro* (7, 53). However, due to its high toxicity and unforeseen drug-drug interactions, all clinical trials of VRP for the reversal of P-gp-mediated MDR failed (53). In addition, the development of other P-gp inhibitors has been disappointing (7). Alternatively, the design of anticancer drugs that are poor P-gp substrates may be an effective strategy to circumvent P-gp-mediated MDR (7, 8). SW480, HCT116 and Caco2 cells are commonly used cell culture models for the study of colorectal cancer *in vitro* (3, 25). QTMP treatment showed cytotoxic activity against SW480 and HCT116 cells, which was similar to that of oxaliplatin treatment in the present study. However, the anticancer activity of QTMP was much better than that of oxaliplatin or paclitaxel in Caco2 cells, a cell line with P-gp overexpression and natural MDR (25). Additionally, QTMP exhibited a potent anticancer activity in SW480 cell-bearing mice. Interestingly, although the mechanisms of drug resistance in HCT116/DDP cells are not clear, QTMP also displayed strong anticancer activity against HCT116/DDP cells. Notably, polymers have large molecular weights and complex spatial structures, which reduces the risk of these compounds becoming P-gp substrates (21–23). VRP did not affect the anticancer activity of QTMP, which suggests that QTMP is not a substrate of P-gp. These findings indicate that QTMP may be an effective anti-CRC drug, including in CRC cells with P-gp-mediated MDR. Importantly, QTMP likely overcame P-gp-mediated MDR because QTMP is not a P-gp substrate.

Accumulation of intracellular ROS may damage DNA, proteins and membranes of organelles, which ultimately activates apoptosis to exert anticancer activity (36, 37). Therefore, induction of oxidative stress by anticancer drugs is an effective therapeutic strategy to kill cancer cells. Similarly, the overproduction of NO, a prominent RNS, causes severe nitrosative stress, which damages organelles and leads to cell injury or death (54). The present findings demonstrated that QTMP induced ROS and RNS production in SW480, HCT116 and Caco2 cells in a concentration-dependent manner. Notably, the ROS scavenger NAC and the NO scavenger hemoglobin inhibited QTMP-induced apoptosis in these CRC cells, indicating that QTMP-induced apoptosis depends on ROS and RNS levels. Further, most studies have reported that overproduction of ROS and RNS can damage mitochondria and activate the mitochondrial apoptotic pathway in cancer cells (55, 56). As expected, the levels of cleaved caspase-9 and Bax, which are marker proteins of the mitochondrial apoptotic pathway, were increased in QTMP-treated CRC cells in a concentration-dependent manner. In addition, the pan-caspase inhibitor z-VAD-FMK significantly inhibited QTMP-induced apoptosis and cell proliferation inhibition in CRC cells. Therefore, these data collectively indicate that QTMP induces apoptosis through ROS- and/or RNS-mediated mitochondrial apoptotic pathways.

DNA is widely used as an anticancer drug target in clinical treatment and research for decades (57, 58). Histone H2AX is phosphorylated at Ser139 (γ-H2AX) immediately after DNA damage (59). The expression of γ-H2AX (Ser139) increased significantly in QTMP-treated SW480, HCT116 and Caco2 cells. However, NAC and hemoglobin reversed QTMP-induced DNA damage in these CRC cells. The positively charged QTMP could combine with the negatively charged DNA *via* electrostatic interactions. The fluorescence intensity of DNA is enhanced after EB insertion into DNA base pairs (25). Similarly, in this study, the fluorescence intensity of the EB-ctDNA complex decreased rapidly with increasing concentrations of QTMP. Moreover, the results of agarose gel electrophoresis revealed that the ct-DNA-QTMP complex samples were gradually retained at the starting position as the QTMP concentration increased because QTMP binding increases the molecular weight of DNA and slows QTMP-DNA complex migration. Therefore, in addition to indirectly damaging DNA by increasing the production of ROS and RNS, QTMP can also directly impact DNA by electrostatic interactions. That is, the resulting ROS and/or RNS overproduction and DNA damage may form a positive feedback loop.

In conclusion, QTMP is a self-assembled nanoparticle with good water solubility, and it is a quaternization of thiourea main-chain polymers. QTMP exhibited potent cytotoxic activity against CRC cells, including SW480, HCT116, Caco2 and HCT116/DDP cells. Meanwhile, it also displayed a strong anticancer activity against SW480 cells *in vivo*. Moreover, as it is not a P-gp substrate, QTMP had a more intense cytotoxic activity towards Caco2 cells, a cell line with P-gp-mediated MDR. In terms of its mechanism of action, in addition to its P-gp avoidance effect, QTMP also caused DNA damage *via* direct insertion into DNA and indirect regulation of ROS and/or RNS production in these CRC cells. As a consequence of overproduction of ROS and RNS, QTMP caused caspase-dependent apoptosis. These results were primarily obtained with *in vitro* assays, and it is important to confirm the results with other study approaches in future research. However, the present study indicates that the design and synthesis of anticancer drugs based on thiourea polymers is promising and valuable and that it offers a new strategy to address MDR. These results provide reference resources for further investigation of thiourea polymers. Therefore, QTMP may be a promising anticancer agent for patients with CRC, including those bearing cancer cells with P-gp-mediated MDR.

## Data Availability Statement

The raw data supporting the conclusions of this article will be made available by the authors, without undue reservation.

## Ethics Statement

The animal study was reviewed and approved by the Experimental Animal Ethic Committee of China Pharmaceutical University and the Science and Technology Department of Jiangsu Province.

## Author Contributions

ZB performed the experiments and data analyses. QZ, HZ and XY participated in the analysis and interpretation of the data. LM and PW participated in the experimental design, conceived the idea, directed the research and contributed to the writing of the manuscript. All authors contributed to the article and approved the submitted version.

## Funding

This work was supported by grants from the National Natural Science Foundation (81903642), the Fifteenth Batch High-level Talents Project of “Six Talent Peaks” in Jiangsu Province (WSW-049), the talents program of Jiangsu Cancer Hospital (YC201809), China Postdoctoral Science Foundation (2020M681528) and Jiangsu Cancer Hospital Postdoctoral Science Foundation (SZL202015).

## Conflict of Interest

The authors declare that the research was conducted in the absence of any commercial or financial relationships that could be construed as a potential conflict of interest.

## Supplementary Material

Supplementary Figure 1
^1^H NMR spectrum for monomer DICH.

Supplementary Figure 2 Gel permeation chromatograph for polymer TMP.

Supplementary Figure 3 The proliferation inhibitory effects of QTMP (0~100 μg/ml) on HCT116/DDP cells were detected by an MTT assay. ***p* < 0.01 *vs* control.

Supplementary Figure 4 Anticancer effects of QTMP in SW480 cell-bearing mice. **(A)** Images of resected xenograft samples. **(B)** Average tumor volumes. **(C)** Average tumor weight. **(D)** The body weights of mice. **(E)** The viscera indexes of mouse main organs. ***p* < 0.01 *vs* the model group.

Supplementary Figure 5
**(A)** FACS analysis for cell cycle distribution after treatment with QTMP (5, 10, 20 μg/ml) for 48 h in HCT116/DDP cells. The original pictures (left); quantification of cell percentages in specific cell cycle phases (right). **(B)** HCT116/DDP cells treated with QTMP (5, 10, 20 μg/ml) for 48 h were stained with Annexin V-FITC/PI double-staining kits to identify the apoptotic cells; quantification (right). ***p* < 0.01 *vs* control.

Supplementary Figure 6 DCFH-DA staining for ROS detection in CRC cells treated with QTMP (5, 10, 20 μg/ml). ***p* < 0.01 *vs* control.

Supplementary Figure 7 DAF-FM DA staining for RNS detection in CRC cells treated with QTMP (5, 10, 20 μg/ml). ***p* < 0.01 *vs* control.

Supplementary Figure 8 Annexin V-FITC/PI double-staining of CRC cells pretreated with hemoglobin (20 μM) or vehicle for 1 h and continually incubated with QTMP (10 μg/ml) for another 48 h. ^##^ *p* < 0.01 *vs* QTMP alone.

Supplementary Figure 9 The cell proliferation inhibition rates were measured by an MTT assay. SW480, HCT116 and Caco2 cells pretreated with hemoglobin (20 μM) or vehicle for 1 h were continually incubated with QTMP (10 μg/ml) for another 48 h. ^##^ *p* < 0.01 *vs* QTMP alone.

Supplementary Figure 10 Cells were treated with paclitaxel alone or in combination with VRP (10 mM) for 72 or 48 h prior to the MTT assay or Annexin V-FITC/PI double-staining. ***p* < 0.01 *vs* control; ^##^ *p* < 0.01 *vs* QTMP alone.
