# Supplementary figures and images for: QTMP, a Novel Thiourea Polymer, Causes DNA Damage to Exert Anticancer Activity and Overcome Multidrug Resistance in Colorectal Cancer Cells
Source: Front Oncol. 2021 May 28;11:667689. doi: 10.3389/fonc.2021.667689 (PMC8194350; doi:10.3389/fonc.2021.667689)

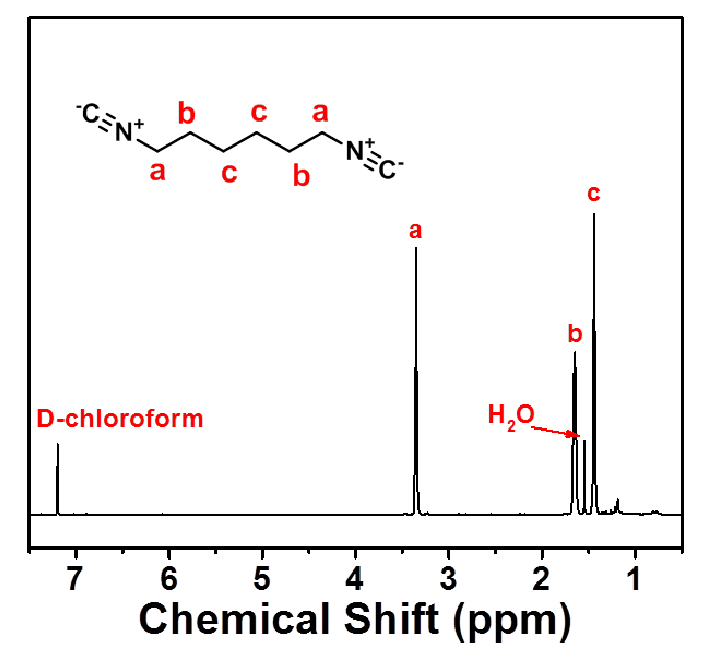

Supplement: Supplementary Figure 1 — 1H NMR spectrum for monomer DICH. [file Image_1.tif]

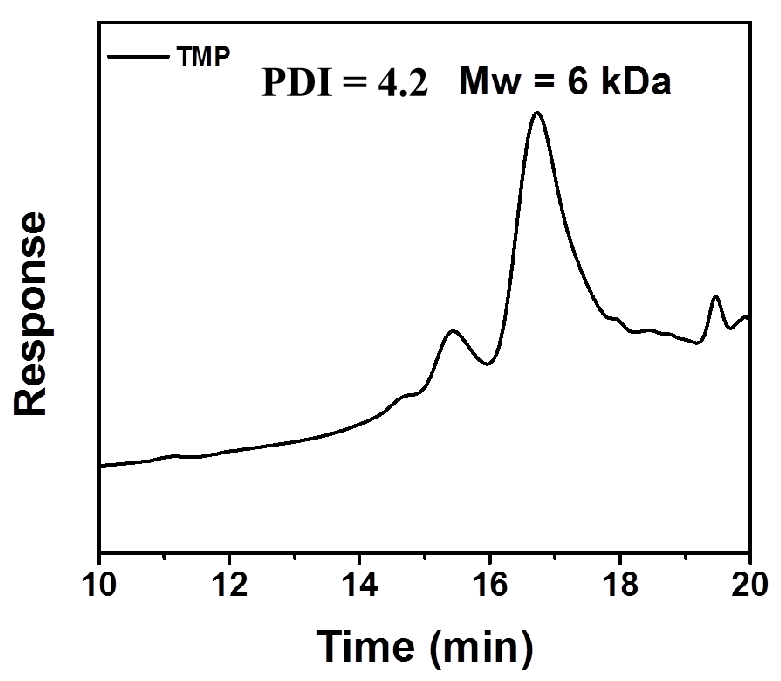

Supplement: Supplementary Figure 2 — Gel permeation chromatograph for polymer TMP. [file Image_2.tif]

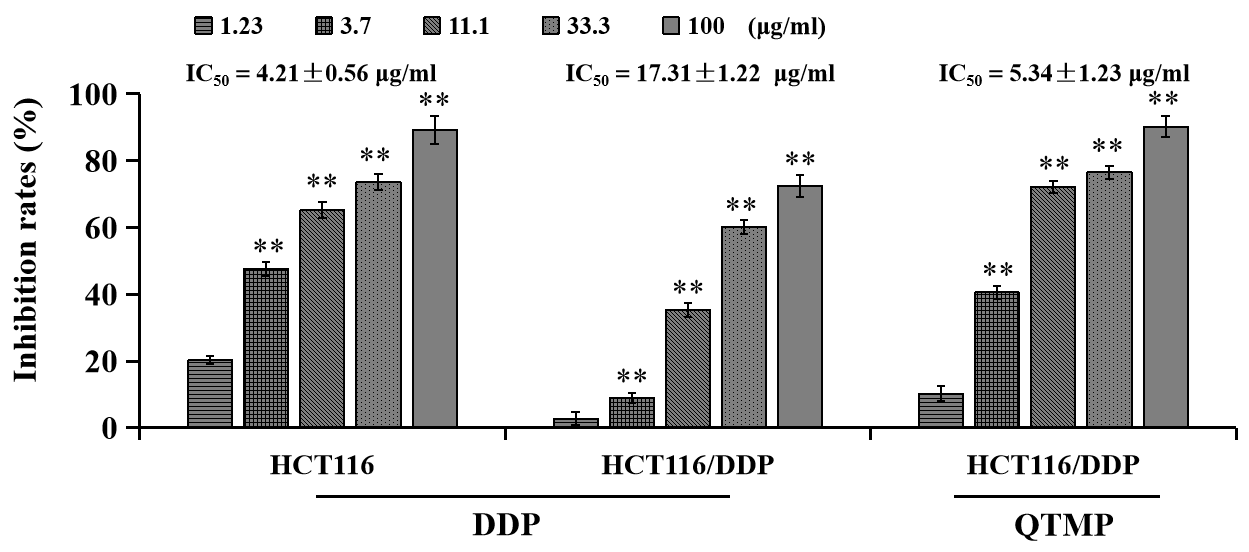

Supplement: Supplementary Figure 3 — The proliferation inhibitory effects of QTMP (0~100 μg/ml) on HCT116/DDP cells were detected by an MTT assay. **p < 0.01 vs control. [file Image_3.tif]

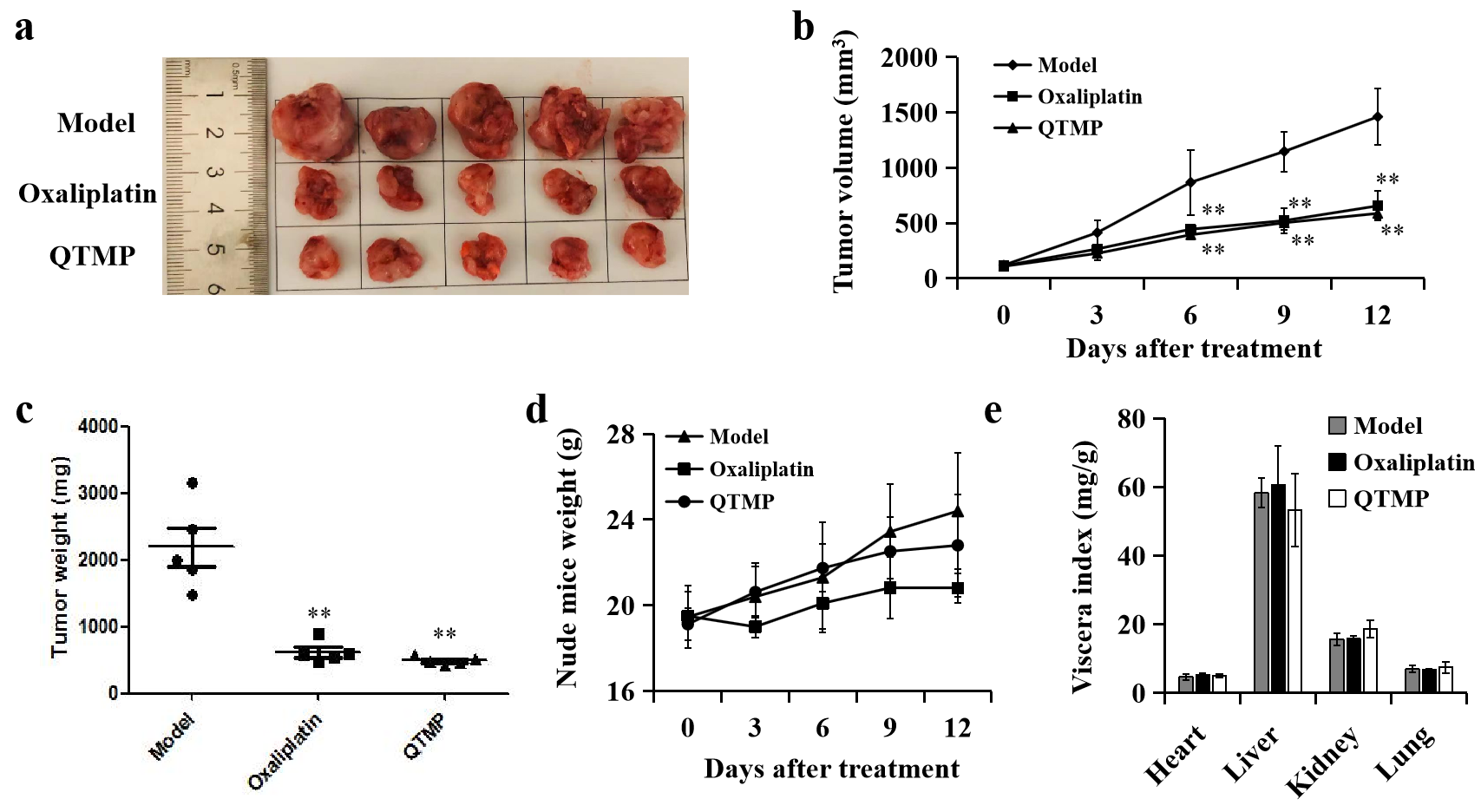

Supplement: Supplementary Figure 4 — Anticancer effects of QTMP in SW480 cell-bearing mice. (A) Images of resected xenograft samples. (B) Average tumor volumes. (C) Average tumor weight. (D) The body weights of mice. (E) The viscera indexes of mouse main organs. **p < 0.01 vs the model group. [file Image_4.tif]

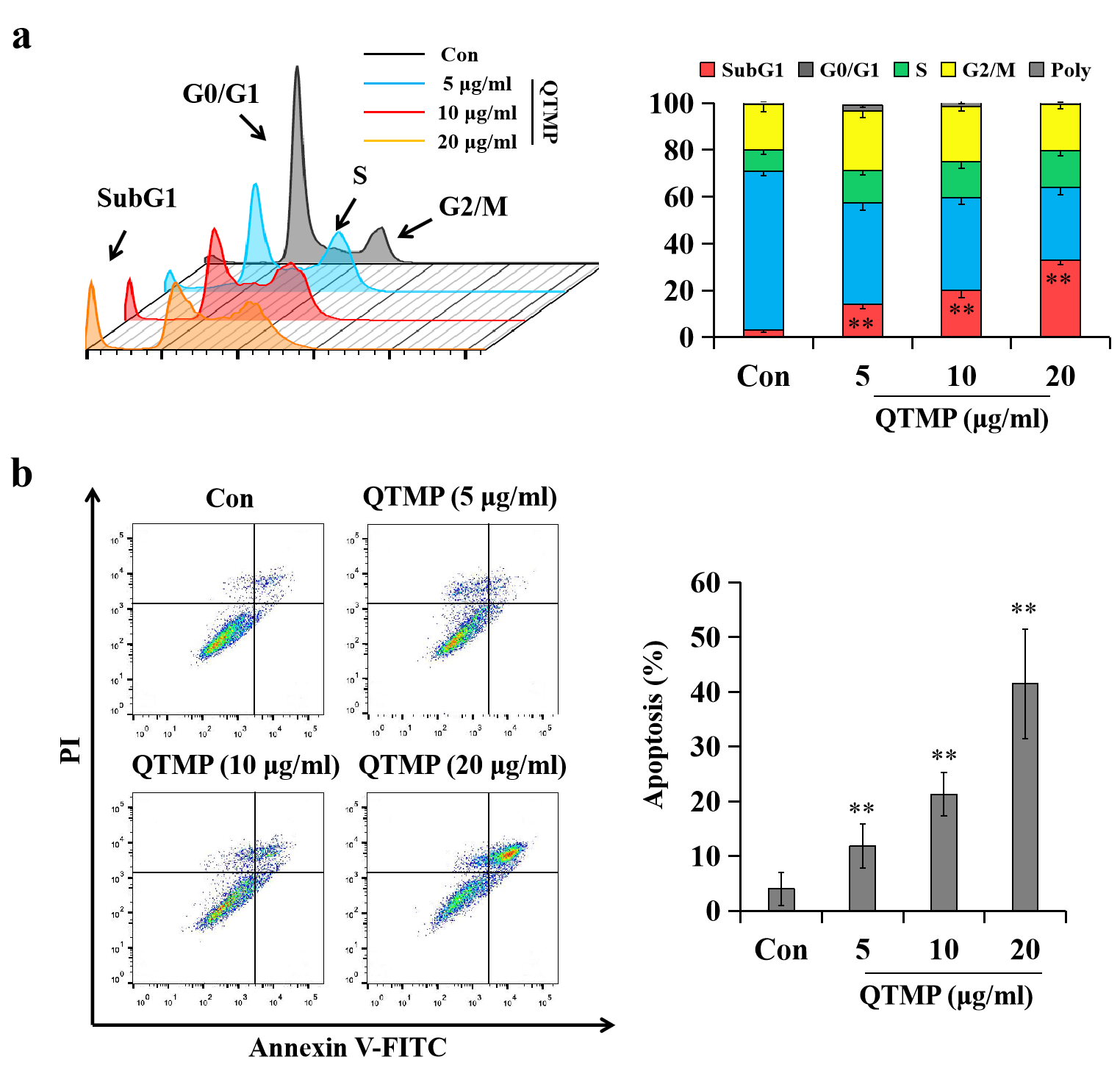

Supplement: Supplementary Figure 5 — (A) FACS analysis for cell cycle distribution after treatment with QTMP (5, 10, 20 μg/ml) for 48 h in HCT116/DDP cells. The original pictures (left); quantification of cell percentages in specific cell cycle phases (right). (B) HCT116/DDP cells treated with QTMP (5, 10, 20 μg/ml) for 48 h were stained with Annexin V-FITC/PI double-staining kits to identify the apoptotic cells; quantification (right). **p < 0.01 vs control. [file Image_5.tif]

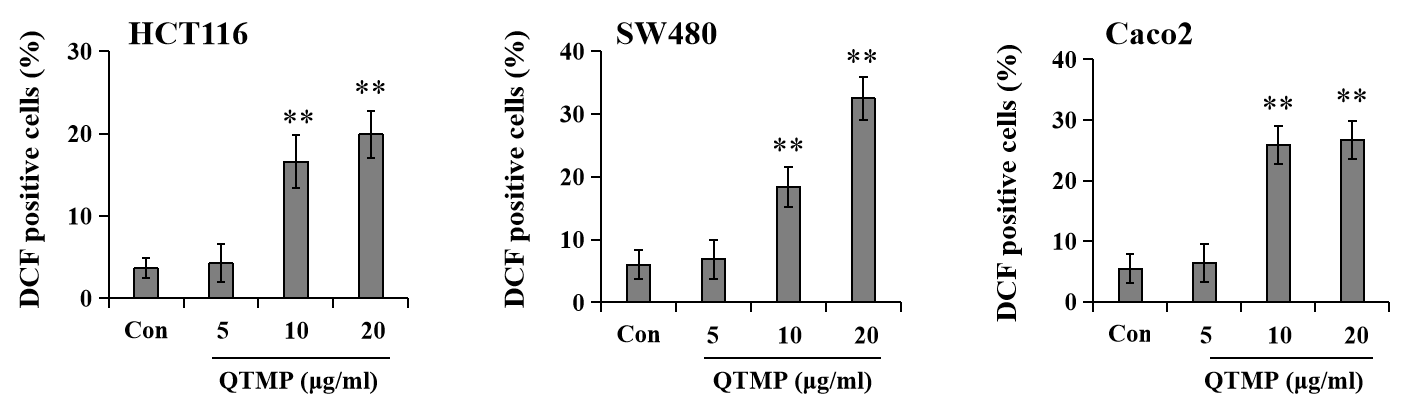

Supplement: Supplementary Figure 6 — DCFH-DA staining for ROS detection in CRC cells treated with QTMP (5, 10, 20 μg/ml). **p < 0.01 vs control. [file Image_6.tif]

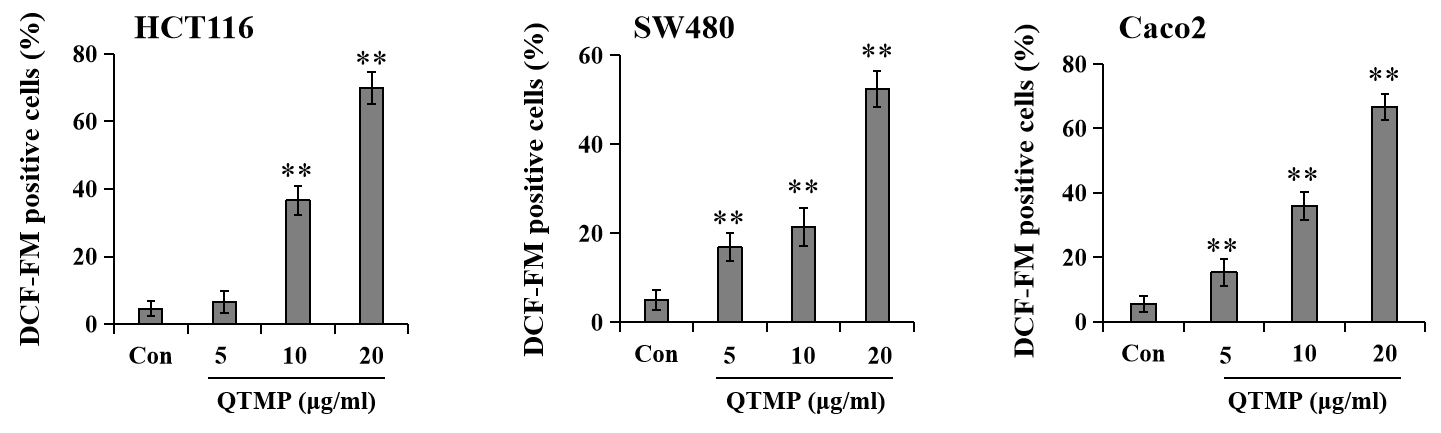

Supplement: Supplementary Figure 7 — DAF-FM DA staining for RNS detection in CRC cells treated with QTMP (5, 10, 20 μg/ml). **p < 0.01 vs control. [file Image_7.tif]

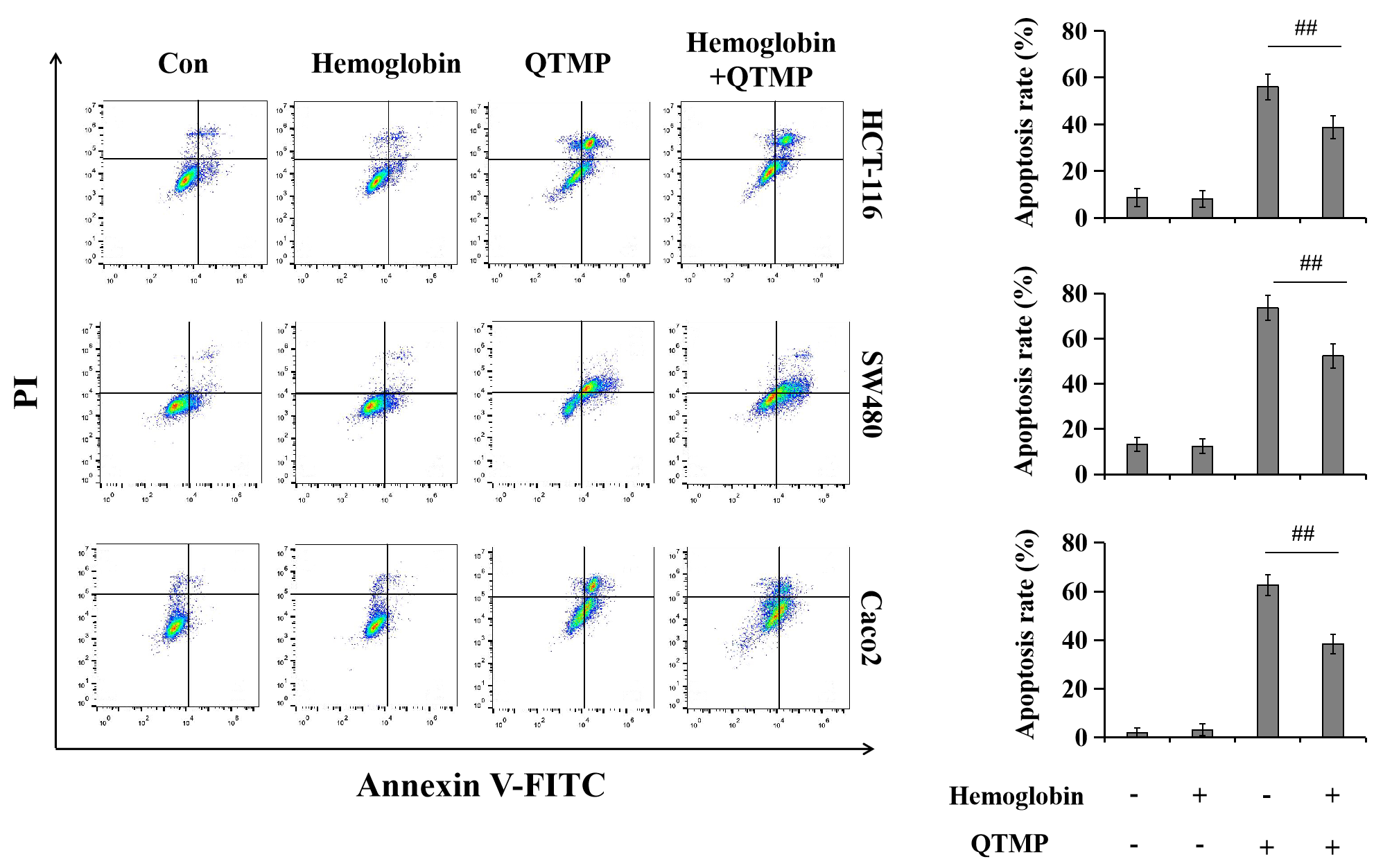

Supplement: Supplementary Figure 8 — Annexin V-FITC/PI double-staining of CRC cells pretreated with hemoglobin (20 μM) or vehicle for 1 h and continually incubated with QTMP (10 μg/ml) for another 48 h. ## p < 0.01 vs QTMP alone. [file Image_8.tif]

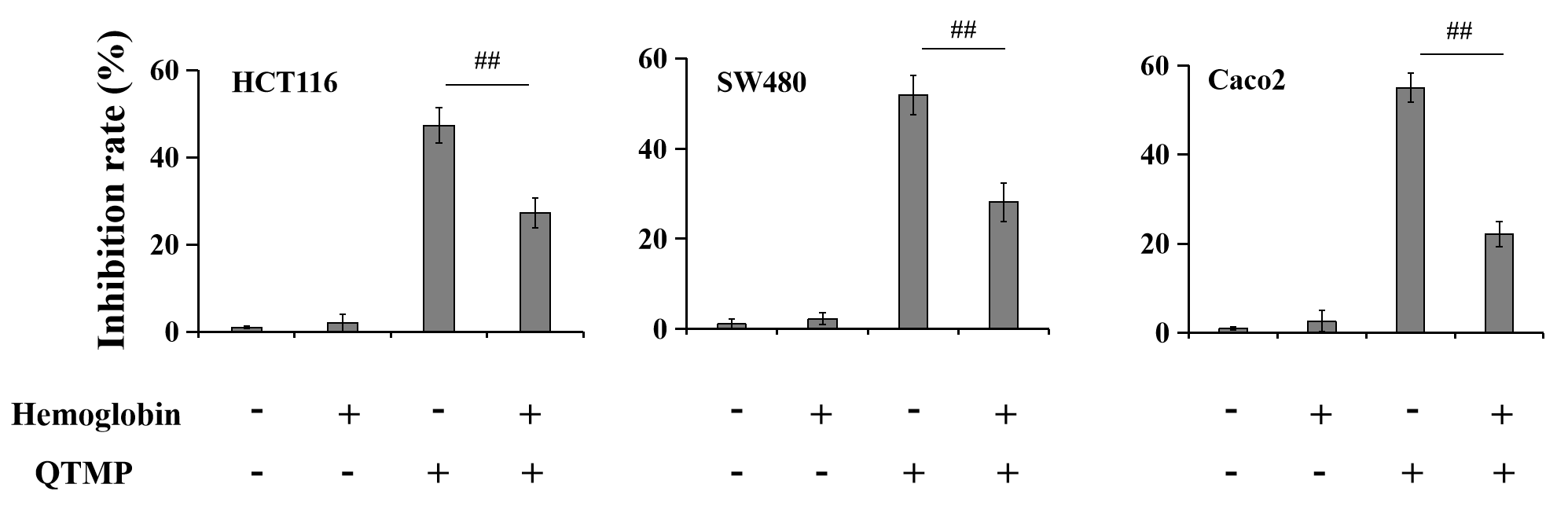

Supplement: Supplementary Figure 9 — The cell proliferation inhibition rates were measured by an MTT assay. SW480, HCT116 and Caco2 cells pretreated with hemoglobin (20 μM) or vehicle for 1 h were continually incubated with QTMP (10 μg/ml) for another 48 h. ## p < 0.01 vs QTMP alone. [file Image_9.tif]

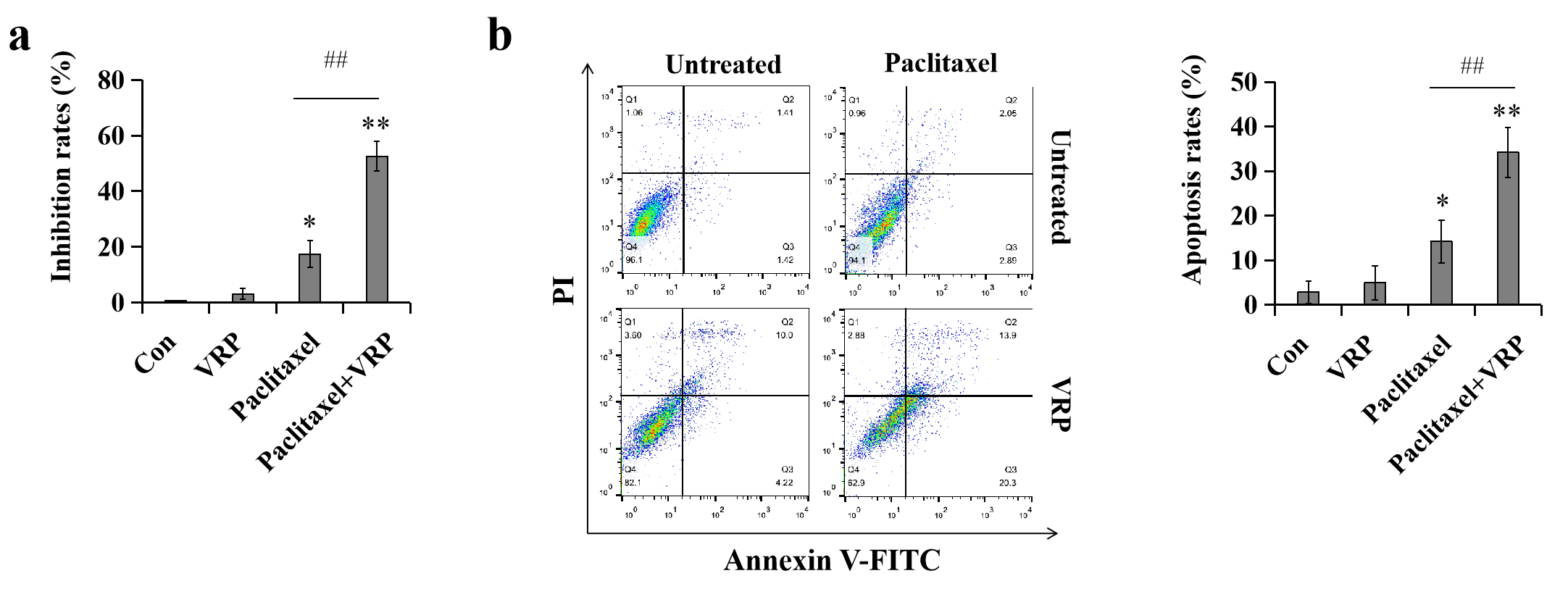

Supplement: Supplementary Figure 10 — Cells were treated with paclitaxel alone or in combination with VRP (10 mM) for 72 or 48 h prior to the MTT assay or Annexin V-FITC/PI double-staining. **p < 0.01 vs control; ## p < 0.01 vs QTMP alone. [file Image_10.tif]
